# Essential consultants’ skills and attitudes (Willing CONSULT): a cross-sectional survey

**DOI:** 10.1186/s12909-021-02810-9

**Published:** 2021-07-03

**Authors:** Takahiro Matsuo, Kuniyoshi Hayashi, Yuki Uehara, Nobuyoshi Mori

**Affiliations:** 1 Department of Infectious Diseases, St. Luke’s International Hospital, 9-1 Akashi-cho, Chuo-ku, Tokyo, Japan; 2 Graduate School of Public Health, St. Luke’s International University, Tokyo, Japan; 3 Department of Clinical Laboratory, St. Luke’s International Hospital, Tokyo, Japan; 4 Department of Microbiology, Juntendo University Faculty of Medicine, Tokyo, Japan; 5 Department of General Medicine, Juntendo University Faculty of Medicine, Tokyo, Japan

**Keywords:** Consultancy, principal component analysis, Exploratory factor analysis

## Abstract

**Background:**

Despite multi-professional collaboration via consultation being increasingly important given the variety of disease diagnoses and treatment, the key elements as consultants remain unclear. The study aimed to identify the skills and attitudes that are important for consultants from the residents’ perspective so that they can be targeted as priority goals in subsequent educational interventions.

**Methods:**

We conducted our research in two phases: a preliminary survey (May 1 to 14, 2020) and a main survey (June 1 to 14, 2020). As a preliminary survey, first-year postgraduate residents at St. Luke’s International Hospital in Tokyo, Japan, were first asked an open-ended question about the types of skills and attitudes that are important for consultants. After eliminating duplicate answers, there were 19 skills and attitudes in total. In the main survey with residents who completed their residency training at our institute, from 2014 to 2018 and current residents (2019–2020), we first asked them about their demographic characteristics (gender, years of postgraduate education, and type of specialty). Then, they answered how important each skill and attitude are for consultants. All 19 items were scored on a seven-point Likert scale that ranged from 0 (completely disagree) to 6 (totally agree). Cronbach’s alpha confirmed the internal consistency of the questionnaire items. Principal component analysis and exploratory factor analysis were performed.

**Results:**

The survey included 107 individuals (61.1 %, 175 potential participants). The median postgraduate years of education was four (interquartile range: 2–5), and 64.5 % were men (*n* = 69). Seven key elements for consultants were identified and termed Willing CONSULT. These included (1) willingness (willingness to accept consultation requests), (2) contact (easy access to consultants), (3) needs (consideration of consulters’ needs), (4) suggestions and support (providing clear recommendations and suggestions, following up on the patients, and supporting the consulters continuously), (5) urgency (considering the situation’s urgency and responding appropriately), (6) learning opportunities (providing teaching points), and (7) text (writing medical records).

**Conclusions:**

We propose Willing CONSULT, which are important skills and attitudes for consultants.

## Background

Interprofessional consultation that provides multiple perspectives is an important aspect of improving the quality of patient care for clinicians [1, 2]. Consultation is defined as a voluntary process in which one professional assists another to address a third party’s problem [3]; in the clinical setting, this usually has patient recovery as the primary goal. Most consultations are requested by the primary team so that they can gain a more specialized perspective on the patient’s care from those with in-depth experience in the specific field. It includes providing professional recommendations, writing notes, following up on patients daily, and teaching residents [1, 2]. In recent years, the number of consultations has increased [4], and there have been numerous reports of the positive association between consultations and favourable patient outcomes, including associations with reduced mortality [5–7], readmission [6, 8], and length of stay [9].

Requests for consultations at training hospitals in Japan are often made formally by residents on the primary team (defined as consulters), and fellows or attending physicians in each specialty receive consultation requests (defined as consultants). This system is similar to that in other countries, such as the United States [10]. Good relationships between residents (consulters) and fellows (consultants) is essential for the patient care [10]. Despite the increasing number of consultations and their importance for patient care, there has been a lack of systematic training in skills as consultant and attitudes that is universal and internationally equivalent [4]. This is important because providing education during the consultation process is often put off due to lack of time or understanding of education methods, and is voluntary. Consultation is a valuable opportunity for both fellows and residents [11]. Several studies have highlighted educational interventions that can improve the quality of resident consultation requests, such as CONSULT by Podolsky et al., [12] and the delivery of resident education by fellows [13]; yet, not many institutes have actively adopted these initiatives. Moreover, previous studies have reported that fellows and residents face challenges in conducting effective consultations in a hospital setting, which include time constraints, differences in team schedules and priorities, and a lack of personal relationships [14–16].

Previous reports of consultant skills include Goldman’s Ten Commandments for Effective Consultations [1], which was published in 1983 and subsequently updated by Salerno [2] in 2007. These updated commandments were (1) determine your customer, (2) establish urgency, (3) look for yourself, (4) be as brief as appropriate, (5) be specific, thorough, and descend from thy ivory tower to help when requested, (6) provide contingency plans and discuss their execution, (7) thou may negotiate joint title to thy neighbour’s turf, (8) teach with tact and pragmatism, (9) talk is essential, and (10) follow up daily. Goldman’s Ten Commandments may be useful for consultants; however, they are based on personal experience rather than an evidence-based recommendation. Moreover, there is a lack of data on the evolution of consulting practices that has resulted from the diversification of physicians’ practices with recent medical advances, and it remains unclear whether these elements apply directly to the current situation. In addition, to the best of our knowledge, little research has been conducted into ways to improve the quality of consultations in Japanese teaching hospitals.

Therefore, we aimed to identify the skills and attitudes that are important as a consultant from the consulter’s perspective so that they can be targeted as priority goals in subsequent educational interventions. This may result in better communication through increased satisfaction from consulters, and quality consultations may lead to improved patient outcomes.

## Methods

### Study setting and participants

We targeted residents who had completed their residency training at St. Luke’s International Hospital in Tokyo, Japan, from 2014 to 2018 and who were current residents in 2019–2020. Our institution is a tertiary-level, 520-bed community teaching hospital in urban Tokyo, which employs 20 to 25 first-year postgraduate residents every year.

**Fig. 1:**
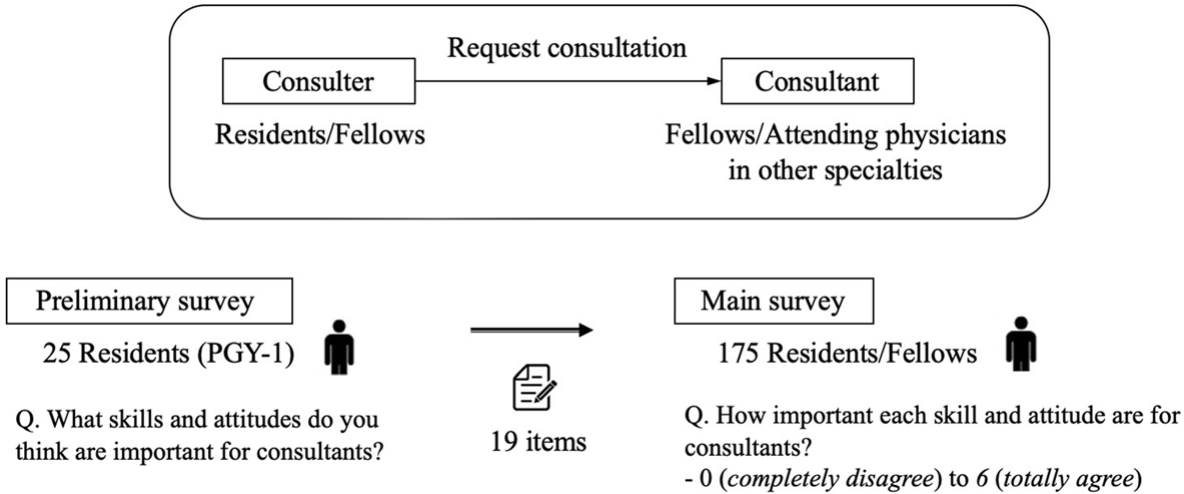
Overview of the process of the consultation and distribution of questionnaires

Requests for research cooperation were emailed to individuals who remained at the hospital during the study period, and those who had moved to other facilities were asked to participate via each year group’s mailing list (Fig. 1). The cover letter informed them that their participation was voluntary and that their responses would remain anonymous, and they were given the uniform resource locators and quick response codes to direct them to the survey. The web-based survey was generated using SurveyMonkey, which is an online survey tool (jp.surveymonkey.com). The completion of the questionnaire implied consent.

### Study design and questionnaire

**Fig. 2:**
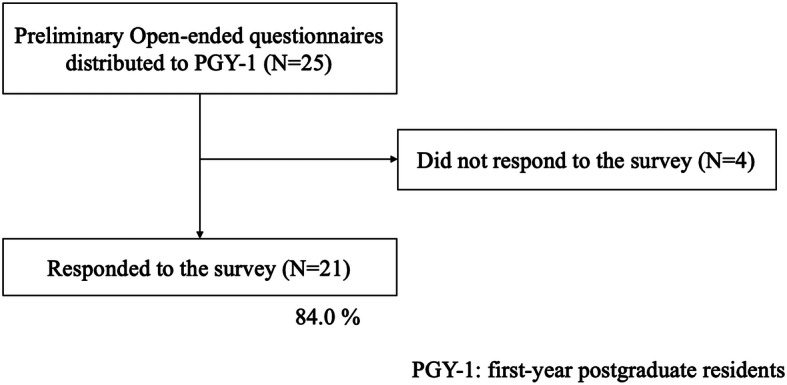
As a preliminary survey, first-year postgraduate residents were first asked an open-ended question about the types of skills and attitudes are important as consultants

**Fig. 3:**
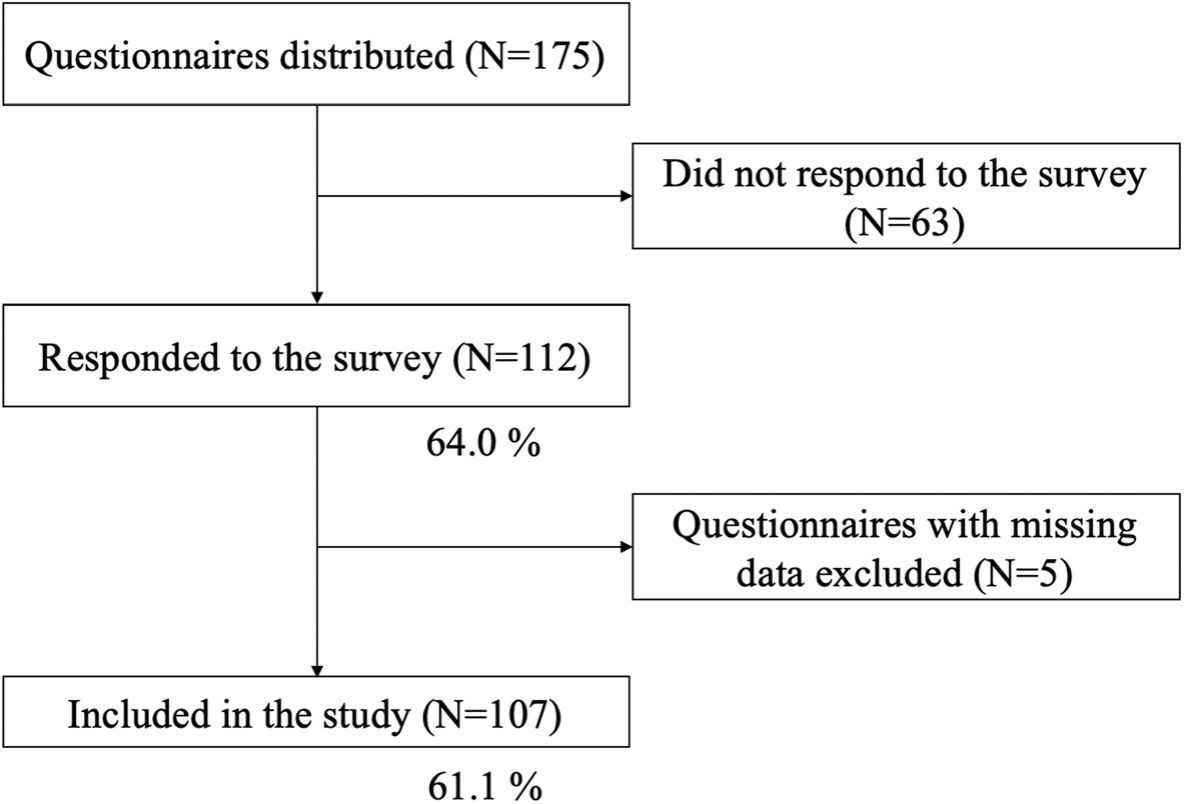
As a main survey, participants answered how important each skill and attitude are for consultants (*N* = 107)

We conducted our research in two phases: a preliminary survey (May 1 to 14, 2020) and a main survey (June 1 to 14, 2020) (Figs. 2 and 3). As a preliminary survey, first-year postgraduate residents were first asked an open-ended question about the types of skills and attitudes that are important for consultants (response rate = 21/25 residents, 84 %) (Fig. 2). In order to conduct factor analysis in the second step of this survey (main survey), we first listed all the skills and attitudes that the participants responded to in the first data collection. Next, we grouped the items that matched the keywords and contents. As a result, 19 different items were extracted. In the main survey with 175 participants, we first asked them about their demographic characteristics (gender, years of postgraduate education, and type of specialty). Then, they answered how important each skill and attitude are for consultants (Fig. 3). All 19 items were scored on a seven-point Likert scale that ranged from 0 (completely disagree) to 6 (totally agree).

### Statistical analyses

First, we calculated Cronbach’s alpha to confirm the internal consistency of the questionnaire items. Then, we used principal components analysis to estimate the appropriate number of factors. We decomposed the correlation matrix that was estimated from the data into eigenvalues and determined the number of factors. After that, we performed an exploratory factor analysis in which we applied the varimax rotation method to the initial estimated factor loadings to interpret each factor. Finally, to determine the validity of the estimated factor model in this exploratory factor analysis, we confirmed the p-value in a statistical hypothesis test to evaluate the model’s goodness of fit to data.

To calculate the sample size in the exploratory factor analysis, it is recommended that the number of participants ranges from 3 to 20 per variable [17]. Our study had 19 elements. Thus, we set the required sample size to approximately 100. Also, to evaluate the sample size calculation quantitatively, we performed the analysis of Kaiser-Meyer-Olkin measure of sampling adequacy. As a result, we got the value of 0.797 on Kaiser-Meyer-Olkin measure of sampling adequacy. Then, it was judged that the sample size for carrying out the factor analysis was obtained in this study. We performed all analyses using SPSS 19.0 J (IBM Japan, Tokyo, Japan) and R version 3.4.1 (R Foundation for Statistical Computing, Vienna, Austria, https://www.R-project.org/) and used the statistical significance of *p* < 0.05 for the two-tailed tests.

### Ethical approval

This study was approved by the Institutional Review Board at St. Luke’s International Hospital in Tokyo, Japan (approval number: 20-R106) on December 8, 2020. This study was conducted in accordance with the Declaration of Helsinki.

## Results

Of the 175 resident physicians, 112 (64 %) responded to the survey (Fig. 2). Five participants were excluded because of missing values related to the analysis. A total of 107 individuals were included in the survey (median postgraduate years of education = 4, interquartile range: 2–5) and 64.5 % were men (*n* = 69). The types of residency programs were as follows; Internal medicine: *n* = 48 (44.4 %), Surgery: *n* = 30 (27.8 %), Obstetrics/Gynecology: *n* = 8 (7.4 %), Pediatrics: *n* = 8 (7.4 %), Emergency medicine: *n* = 3 (2.8 %), Others: *n* = 10 (9.3 %) (Table 1). Table 2 shows the median scores for each question.

**Table 1:** Participants’ demographics

Participants *N = 107*	
Men, n (%)	69 (64.5)
Post graduate years of education, median (IQR)	4 (2–5)
The types of residency programs, n (%)	
Internal medicine	48 (44)
Surgery	30 (2.8)
Obstetrics/Gynecology	8 (7.4)
Pediatrics	8 (7.4)
Emergency medicine	3 (2.8)
Others	10 (9.3)

**Table 2:** Skills and attitudes required in consultants according to residents

Items	Mean score (SD)
Easy access to consultants (connected by telephone when requesting a consultation)	5.29 (0.952)
Willing to accept consultation requests	5.76 (0.473)
Communicate with the consulters courteously	5.24 (0.920)
Do not show how busy consultants are	4.44 (1.268)
No emotional fluctuations	5.20 (1.103)
Consider the urgency of the situation and respond appropriately	5.17 (0.956)
Consider the needs of the consulters	5.20 (0.976)
Examine the patients together	4.26 (1.119)
Meet with the consulters in person to discuss the issue	4.32 (1.256)
Share the thoughts leading up to the recommendation	5.40 (0.738)
Provide clear recommendations and suggestions	5.63 (0.666)
Provide teaching points	4.77 (1.069)
Provide feedback on the presentation	4.23 (1.186)
Cite the literature and provide recommendations	4.21 (1.229)
Help with orders	2.95 (1.397)
Write the medical records quickly	4.35 (1.222)
Write the medical records in a way that is easy for non-professionals to understand	5.01 (1.023)
Create an atmosphere in which consulters can easily ask questions	5.36 (0.838)
Follow up the patients and support the consulters continuously	5.17 (0.916)

All 19 items were scored on a seven-point Likert scale ranging from 0 (*completely disagree*) to 6 (*totally agree*)

Abbreviations: *SD *standard deviation

### Cronbach’s alpha and estimation of the number of factors

Cronbach’s alpha was 0.86, which indicated that the internal consistency of the questionnaire items was high. We obtained six eigenvalues that were larger than one. When using Kaiser’s cut-off criterion, we found that there were six factors. However, the cumulative proportion in terms of the eigenvalue was greater than 0.7 for the seventh eigenvalue, and we found that the optimal number of factors was seven when using Jolliffe’s cut-off criterion. By considering the above facts, we selected Jolliffe’s cut-off criterion and set the optimal number of factors at seven.

### Interpretation of the factors via factor analysis

We present the results of the factor analysis with seven factors and varimax rotation in Table 3. We interpreted each factor based on the magnitude of the absolute values of the estimated factor loadings. For the first factor, the magnitude of the absolute values of ‘provide feedback on the presentation’, ‘provide teaching points’, ‘share the thoughts leading up to the recommendation’, and ‘cite the literature and provide recommendations’ was large. We named this first factor ‘learning opportunities’ For the second factor, the magnitude of the absolute values of ‘willing to accept consultation requests’, ‘no emotional fluctuations’, and ‘communicate with the consulters courteously’ was large. We labelled this factor ‘willingness’. For the third factor, the magnitude of the absolute value of ‘consider the urgency of the situation and respond appropriately’ was large, and we named this ‘urgency’. Regarding the fourth factor, the magnitude of the absolute values of ‘provide clear recommendations and suggestions’ and ‘follow up the patients and support the consulters continuously’ was large. We called this ‘suggestions/support’. For the fifth factor, the magnitude of the absolute values of ‘write the medical records quickly’ and ‘write the medical records in a way that is easy for non-professionals to understand’ was large. We labelled this factor ‘text’. Regarding the sixth factor, the magnitude of the absolute value of ‘consider the needs of the consulters’’ was large, and we named this ‘needs”. For the seventh factor, the magnitude of the absolute value of ‘easy access to consultants (connected by telephone when requesting a consultation)’ was large. We called this ‘contact’ (Table 4).

**Table 3:** Factors identified by factor analysis (with varimax rotation)

Items	Factor 1	Factor 2	Factor 3	Factor 4	Factor 5	Factor 6	Factor 7
Easy access to consultants (connected by telephone when requesting a consultation)	-0.028	0.196	0.215	0.331	-0.035	0.230	0.551
Willing to accept consultation requests	0.085	0.581	0.288	0.045	-0.160	-0.077	0.169
Communicate with the consulters courteously	0.245	0.674	0.154	0.080	0.083	0.146	0.035
Do not show how busy consultants are	0.162	0.106	0.438	0.477	-0.112	-0.006	0.074
No emotional fluctuations	0.095	0.730	0.172	0.271	0.025	0.156	-0.026
Consider the urgency of the situation and respond appropriately	0.117	0.152	0.551	0.423	0.243	0.178	0.182
Consider the needs of the consulters	-0.085	0.554	0.297	0.171	0.297	0.786	0.263
Examine the patients together	0.551	0.181	0.387	0.177	-0.011	0.150	0.108
Meet with the consulters in person to discuss the issue	0.439	0.162	0.278	0.168	0.006	0.173	0.224
Share the thoughts leading up to the recommendation	0.680	0.186	-0.185	-0.047	0.119	-0.007	0.399
Provide clear recommendations and suggestions	0.068	0.184	0.110	0.926	0.224	0.074	-0.026
Provide teaching points	0.888	0.135	0.148	0.048	-0.014	-0.030	-0.118
Provide feedback on the presentation	0.769	0.122	0.273	0.076	0.051	0.077	-0.026
Cite the literature and provide recommendations	0.785	-0.019	0.154	0.143	0.065	-0.079	0.037
Help with orders	0.237	0.383	0.504	0.216	0.145	0.124	-0.160
Write the medical records quickly	0.111	0.112	0.507	0.242	0.520	0.049	0.191
Write the medical records in a way that is easy for non-professionals to understand	0.063	0.147	0.219	-0.021	0.690	0.191	0.102
Create an atmosphere in which consulters can easily ask questions	0.531	0.397	-0.145	-0.151	0.346	0.032	0.180
Follow up the patients and support the consulters continuously	0.142	0.060	0.247	0.514	0.341	-0.083	0.139

**Table 4:** The factors of Willing CONSULT and the related skills and attitudes required in consultants

Factors	
Willingness	Willing to accept consultation requests/communicate with the consulters courteously/no emotional fluctuations
Contact	Easy access to consultants (connected by telephone when requesting a consultation)
Needs	Consider the needs of the consulters
Suggestions/support	Provide clear recommendations and suggestions, follow up the patients, and support the consulters continuously
Urgency	Consider the urgency of the situation and respond appropriately
Learning opportunities	Provide teaching points/provide feedback on the presentation/share the thoughts leading up to the recommendation/cite the literature and provide recommendations
Text	Write the medical records in a way that is easy for non-professionals to understand/write the medical records quickly

### Validity of the seven-factor model

Our analysis revealed that this seven-factor model was an appropriate fit for the dataset obtained (*p* = 0.84). Therefore, we considered the above seven factors to be sufficient.

## Discussion

In our study, we summarized the important skills and attitudes of consultants in a teaching hospital, particularly from the perspective of the resident doctors. By using a questionnaire, we statistically identified seven key elements for consultants, which we called Willing CONSULT. We included (1) willingness (willing to accept consultation requests), (2) contact (easy access to consultants), (3) needs (consideration of the needs of the consulters), (4) suggestions and support (providing clear recommendations and suggestions, following up on patients, and supporting the consulters continuously), (5) urgency (considering the urgency of the situation and responding appropriately), (6) learning opportunities (providing teaching points), and (7) text (writing the medical records) (Table 4). Furthermore, we have newly identified that willingness and easy access to consultants are thought to be important for consultants. Notably, most participants indicated that willingness was the most important element for consultants (median = 5.7/6). In other words, the consultant’s attitude toward the initial request for consultation was the most important factor for residents during the consultation process, rather than the content of the education or the recommendations. Studies have reported that the fellows’ resistance or high-handedness when receiving requests for consults can promote the residents’ anxiety [18] and can be a barrier to good communication. Moreover, pushback to a consultation request, which is defined as reluctance or resistance to conducting a consultation, has a negative impact on the resident-fellow relationship [14]. Specific examples of pushback include ‘the consultation was not necessary’, ‘it should have been called for earlier in the day’, ‘a different service should have been requested’, ‘the consultation questions were not clear’, and ‘the information presented by the resident was insufficient’. Furthermore, the tone of voice when receiving a request for a consultation can also be interpreted as pushback [14]. Residents may withdraw the request for a consultation with fellows due to the difficulty. Therefore, because delays in consultation can have a negative impact on patient care [9], consultants should be aware that they need to demonstrate a willingness to accept consultation requests.

Our findings also provide a new perspective on the importance of easy access to consultants when conducting consultations. Whereas a smooth consultation process enables subsequent discussions and management decisions, delays can lead to unfavourable patient outcomes [9]. It is easy to imagine how stressful it is for residents when a consultation is urgent, especially if a consultant is responding to another call or performing a procedure or surgery. Although each hospital and country use different consultation methods, such as via pager, email, telephone calls, and request letters in medical records [19–21], the same issues may apply in facilities with systems that are similar to ours, which requires a telephone call for consultation requests. Recent studies have revealed that consultation via secure messaging applications can be useful for timely medical care and information sharing [22] and may be effective for achieving quick and productive consultations. Other key elements–(1) considering the needs of the consulters, (2) providing clear recommendations and suggestions, (3) following up on patients and supporting the consulters continuously, (4) considering the urgency of the situation and respond appropriately, (5) providing teaching points, and (6) writing the medical records–were similar to those in previous reports, such as Kesslers’ 5Cs (Contact, Communicate, Core question, Collaboration, and Closing the loop) [23], Chen’s PARTNER (Partner with resident, Assess the learner, Reinforce positives, Teaching objectives, New knowledge, Execute recommendations, and Review) [24], Chan’s PIQUED (Prepare, Identify, Question, Urgency, Educational modifications, Debrief) [25], and the Ten Commandments for Effective Consultations [1, 2]. The education component is particularly essential when residents have many opportunities to consult with fellows in teaching hospitals, as in our study. Certainly, it is challenging for fellows to strike a balance between inpatient service and resident education [26]. With these issues in mind, the problems of time and schedules cannot be solved by consultants acquiring skills alone, and structural changes in support are necessary. However, teaching during consultation may have a positive impact on patient care through increased collaboration and building relationships [1, 2]. The results of this study could influence aspects of consultant attitudes and appropriate behaviors, suggesting the possibility of improvement by consultants themselves and consultant training methods. Moreover, a consultant’s work rarely ends with an initial consultation. Appropriate follow-up visits that are documented in the medical records could create a trusting relationship and improve the compliance of the primary team [27].

Our study has several limitations. First, as it was conducted in a single institution, our data cannot be generalized to different institutions and countries where there are different consultation systems. Second, our survey included only young residents. Our hospital is characterized by the fact that residents who are rotating in a department mostly provide the consultations, rather than fellows or attending physicians. Therefore, although we found that the education component is important, it should be noted that this may not apply if the request is made by fellows or attending physicians. Finally, we included the former residents who completed their residency in addition to current residents. Since former residents are now fellows or consultants at the time of the survey, we cannot deny the possibility of bias due to a change in mindset. Multicenter studies including only current residents are warranted to confirm these findings.

One strength of our study is that we newly identified the key elements for consultants based on the data analysis, which covers the entire consultation process from the beginning to the follow up. Our study suggests that these elements could be effective in teaching hospitals, particularly when consults are provided by younger residents to fellows or attending physicians. Moreover, we based our Willing CONSULT framework, which contains seven easy-to-remember key elements, on direct responses from residents as the consulters. Therefore, in addition to the traditional ten commandments, the new elements of willingness and easy access to consultants have the potential to further increase the residents’ satisfaction and improve the quality of the consultation.

## Conclusions

In our study, we proposed Willing CONSULT, which consists of seven key elements that are important skills and attitudes for consultants. Our findings indicate that further research investigating its external validity by conducting multicentre studies and examining whether educational interventions that focus on these elements is needed to improve the quality of consultations and patient outcomes

## Data Availability

The datasets generated and/or analysed during the current study are not publicly available due the dataset overlaps with another ongoing project and we need permission from our institution to obtain the dataset, but are available from the corresponding author on reasonable request.

## References

[1] Goldman L, Lee T, Rudd P (1983). Ten commandments for effective consultations. Arch Intern Med.

[2] Salerno SM, Hurst FP, Halvorson S, Mercado DL (2007). Principles of effective consultation. Arch Intern Med.

[3] Lytle RK, Collier D (2002). The consultation process: Adapted physical education specialists’ perceptions. Adapt Phys Act Q.

[4] Serling-Boyd N, Miloslavsky EM (2020). Enhancing the inpatient consultation learning environment to optimize teaching and learning. Rheum Dis Clin North Am.

[5] Ishikane M, Hayakawa K, Kutsuna S, Takeshita N, Ohmagari N (2019). The impact of infectious disease consultation in candidemia in a tertiary care hospital in Japan over 12 years. PLoS One.

[6] Barkley JE, McCall A, Maslow AL, Skudlarska BA, Chen X (2019). Timing of palliative care consultation and the impact on thirty-day readmissions and inpatient mortality. J Palliat Med.

[7] Liu P, Quinn RR, Karim ME, Bello A, Tam-Tham H, Weaver R (2019). Nephrology consultation and mortality in people with stage 4 chronic kidney disease: A population-based study. CMAJ.

[8] Marks LR, Munigala S, Warren DK, Liang SY, Schwarz ES, Durkin MJ (2019). Addiction medicine consultations reduce readmission rates for patients with serious infections from opioid use disorder. Clin Infect Dis.

[9] Rahman AS, Shi S, Meza PK, Jia JL, Svec D, Shieh L (2019). Waiting it out: Consultation delays prolong in-patient length of stay. Postgrad Med J.

[10] Miloslavsky EM, Criscione-Schreiber LG, Jonas BL, O’Rourke KS, McSparron JI, Bolster MB (2016). Fellow as teacher curriculum: Improving rheumatology fellows’ teaching skills during inpatient consultation. Arthritis Care Res.

[11] Winn AS, Stafford DEJ, Miloslavsky EM, McSparron JI, Grover AS, Boyer D (2019). Making the consult interaction more than a transaction: Helping fellows be better teachers and residents be better learners. J Pediatr.

[12] Podolsky A, Stern DT, Peccoralo L (2015). The courteous consult: A CONSULT card and training to improve resident consults. J Grad Med Educ.

[13] Miloslavsky EM, Degnan K, McNeill J, McSparron JI (2017). Use of fellow as clinical teacher (FACT) curriculum for teaching during consultation: Effect on subspecialty fellow teaching skills. J Grad Med Educ.

[14] Miloslavsky EM, McSparron JI, Richards JB, Puig A, Sullivan AM (2015). Teaching during consultation: Factors affecting the resident–fellow teaching interaction. Med Educ.

[15] Chan T, Bakewell F, Orlich D, Sherbino J (2014). Conflict prevention, conflict mitigation, and manifestations of conflict during emergency department consultations. Acad Emerg Med.

[16] Lingard L, McDougall A, Levstik M, Chandok N, Spafford MM, Schryer C (2012). Representing complexity well: A story about teamwork, with implications for how we teach collaboration. Med Educ.

[17] Loewen S, Gonulal T, Plonsky L (2015). Exploratory factor analysis and principal components analysis. Advancing quantitative methods in second language research.

[18] Baylis J, Miloslavsky EM, Woods R, Chan TM (2019). Conquering consultations: A guide to advances in the science of referral–consultation interactions for residency education. Ann Emerg Med.

[19] Chu CD, Tuot DS, Harrison JD, Duong J, Luxenberg A, Khanna RR (2020). Completeness and quality of text paging for subspecialty consult requests. Postgrad Med J.

[20] Boulware DR, Dekarske AS, Filice GA (2010). Physician preferences for elements of effective consultations. J Gen Intern Med.

[21] O’Connor C, Friedrich JO, Scales DC, Adhikari NKJ (2009). The use of wireless e-mail to improve healthcare team communication. J Am Med Informatics Assoc.

[22] Gulacti U, Lok U (2017). Comparison of secure messaging application (WhatsApp) and standard telephone usage for consultations on length of stay in the ED: A prospective randomized controlled study. Appl Clin Inform.

[23] Kessler CS, Afshar Y, Sardar G, Yudkowsky R, Ankel F, Schwartz A (2012). A prospective, randomized, controlled study demonstrating a novel, effective model of transfer of care between physicians: The 5 Cs of consultation. Acad Emerg Med.

[24] Chen DC, Miloslavsky EM, Winn AS, McSparron JI (2018). Fellow as clinical teacher (FACT) curriculum: Improving fellows’ teaching skills during inpatient consultation. MedEdPORTAL.

[25] Chan T, Orlich D, Kulasegaram K, Sherbino J (2013). Understanding communication between emergency and consulting physicians: A qualitative study that describes and defines the essential elements of the emergency department consultation-referral process for the junior learner. . CJEM.

[26] Backes C, Reber KM, Trittmann JKB (2011). Fellows as teachers: A model to enhance pediatric resident education. Med Educ Online.

[27] Cohn SL (2003). The role of the medical consultant. Med Clin North Am.

